# AIDS in Asia: A Continent in Peril

**DOI:** 10.3201/eid1204.060013

**Published:** 2006-04

**Authors:** Elizabeth Pisani

**Affiliations:** * London School of Hygiene and Tropical Medicine, London, United Kingdom

**Keywords:** AIDS, Asia, HIV

Susan Hunter's book, AIDS in Asia: A Continent in Peril, tackles an important subject. The subheading leaves no doubt that this volume belongs squarely in the "next wave" school—Africa has been devastated by the AIDS epidemic, and Asia must be next. Hunter's book paints a picture of a continent about to be engulfed in social, economic, and political chaos, all because of 1 small retrovirus.

Unfortunately, Hunter presents no data that support this hypothesis. Her chapter on the epidemiology of HIV in Asia, The Looming Mushroom Clouds of Infection, relies heavily on a single report by Nicholas Eberstadt, a demographer and foreign policy analyst who has little experience in the modeling of sexually transmitted and infectious disease. She draws occasionally on advocacy reports by other international organizations such as The Joint United Nations Programme on HIV/AIDS. The book is poorly referenced and shows no evidence that Hunter has attempted to examine surveillance data (much of which is in public domain) or other original data sources. For example, she provides no source for the claim that "HIV… typically infects 50%–90% of a developing country's sex workers." Five years of systematic review of primary surveillance data from thousands of sentinel sites throughout Asia has shown only 1 site with rates >50% among sex workers, Mumbai in India. Virtually all the sex worker sites in the Philippines, Indonesia, China, Bangladesh, Pakistan, the most populous countries in the region other than India, report prevalence rates <5%.

The book is consistently structured. Each chapter begins with conversations between the downtrodden victims of the HIV epidemic in Asia, persons whose clothing, gestures, and thoughts Hunter describes in heart-rending detail, although they are not, in fact, real persons. Next comes a homily based on the life of Emily Pankhurst, the 19th century English feminist whose relevance to the current HIV epidemic in Asia is uncertain. Then comes a bit of Asian history, a nicely written précis of the Mongol advance or the opium wars, for example, and some observations on historical and cultural injustices of life, injustices that ultimately lead people to have unprotected sex or share needles. Each chapter closes with an apocalyptic statement, along the lines of "More and more countries in Asia are teetering on the brink of a violent explosion… and the outcome may be an epidemic the likes of which has never been seen in world history."

Hunter may perhaps be forgiven for absorbing the alarmist tone of the advocacy reports she has read, but more attention to the facts would show that this sort of doom mongering is contradicted by every reliable measure of HIV prevalence and sexual behavior in Asia. These data suggest that in Asia (as in the industrial world), HIV will largely be confined to populations with well-defined risk of exposure to the virus. Hunter is absolutely correct in taking Asian governments to task for not doing enough to confront HIV. However, suggesting that HIV is an impending catastrophe that requires turning Asian societies upside down is unhelpful and outdated. This may be true in Africa, where Hunter has more experience, but had she looked beyond the hype at any real data, Hunter would have found that HIV infection does not, in fact, threaten to engulf Asia in social and economic chaos. It is a relatively well-contained infectious disease that, for prevention and control, requires clean needles, condoms, lubricant, screening for sexually transmitted infections, and treatment for a small proportion of the population. Those goals can, and should, be achieved shortly.

